# Investigation of the potential regulator proteins associated with the expression of major surface protein and dentilisin in *Treponema denticola*

**DOI:** 10.1080/20002297.2020.1829404

**Published:** 2020-10-11

**Authors:** Yuki Arai, Yuichiro Kikuchi, Kazuko Okamoto-Shibayama, Eitoyo Kokubu, Seikou Shintani, Kazuyuki Ishihara

**Affiliations:** a Department of Pediatric Dentistry, Tokyo Dental College, Tokyo, Japan; b Department of Microbiology, Tokyo Dental College, Tokyo, Japan

**Keywords:** *Treponema denticola*, DNA-binding protein, gene expression, dentilisin, major surface protein, repressor

## Abstract

**Objective***Treponema denticola* is involved in ‘chronic’ periodontitis pathogenesis. The mechanism underlying the regulation of the expression of its virulence factors, such as major surface protein (Msp) and prolyl-phenylalanine specific protease (dentilisin) is yet to be clarified. We determined the gene expression profiles of Msp- and dentilisin-deficient mutants of *T. denticola* to identify the regulation network of gene expression concomitant with the inactivation of these virulence genes.

**Methods** Gene expression profiles of *T. denticola* ATCC 35405 (wild type), dentilisin-deficient mutant K1, and *msp-*deficient mutant DMSP3 were determined using DNA microarray analysis and quantitative real-time reverse transcription PCR (qRT-PCR). Msp and dentilisin protein levels were determined by immunoblotting and proteolytic activity assays.

**Results** In addition to several differentially expressed genes, dentilisin expression was reduced in DMSP3; *msp* expression was significantly reduced in K1 (p < 0.05), both at the gene and protein levels. To identify the regulatory system involved, the expression levels of the potential regulators whose expression showed changes in the mutants were evaluated using qRT-PCR. Transcriptional regulators TDE_0127 and TDE_0814 were upregulated in K1, and the potential repressor, TDE_0344, was elevated in DMSP3.

**Conclusions** Dentilisin and Msp expression were interrelated, and gene expression regulators, such as TDE_0127, may be involved in their regulation.

## Introduction

Chronic periodontitis is an infectious disease that leads to the destruction of the periodontal tissues and represents a major cause of tooth loss in adults [1]. Dysbiosis in the subgingival microbiome is a major cause of periodontitis [2]. Along with *Porphyromonas gingivalis* and *Tannerella forsythia*, the prevalence of *Treponema denticola* reportedly increases in the subgingival microbiome during the dysbiotic shift associated with the development of chronic periodontitis [3,4]. This shift is believed to play an important role in the onset and progression of the disease [5]. Further, *T. denticola* was also detected in lesions of apical periodontitis [6]. These reports suggest that *T. denticola* is involved in the dysbiotic shift of the microbiome at the site of inflammation. In addition, *T. denticola* was also detected in systemic diseases such as atherosclerotic lesions [7].

Major surface protein (Msp) and prolyl-phenylalanine-specific protease (dentilisin) are the major virulence factors of *T. denticola* [8]. Dentilisin and Msp are located in the outer sheath of *T. denticola* [9]. Msp is a highly immunogenic 53 kDa protein [10] and organizes a heat modifiable high molecular-weight complex [11]. The structure of Msp, predicted from its amino acid sequence, was reported to be similar to those of other bacterial porins, in which several membrane-spanning amphipathic β sheets are arranged in a pore-forming β barrel [12]. Msp has been reported to possess cytopathic activity owing to its pore-forming activity against epithelial cells [9]. It also mediates the extracellular matrix binding activity of *T. denticola* via adherence to fibronectin and laminin [13].

Dentilisin is a prolyl-phenylalanine-specific protease [14]. It consists of four proteins coded by three genes (*prcB, prcA*, and *prtP*) [14,15]. Among them, *prtP* codes for a 72 kDa protease domain, which degrades components of the extracellular matrix such as fibronectin, activates C3, and is involved in adherence to fibrinogen [14,16,17]. Additionally, dentilisin is also involved in adherence to fibronectin, and immunomodulation [8]. Further, the coaggregation activity between *T. denticola* and *T. forsythia* was reported to be reduced in a dentilisin-dependent manner in the dentilisin-deficient mutant, K1 [18]. Additionally, K1 showed attenuated invasiveness to gingival epithelial cells compared to the wild type strain [19]. Moreover, the gingival epithelial cells infected by *T. denticola* wild type strain ATCC 35405 showed attenuated migration activity, while a lower attenuation was observed with K1 and the Msp-deficient mutant, DMSP3.

Msp and dentilisin have been co-isolated from the outer sheath of *T. denticola* [9], and an oligomeric form of Msp, which was observed in the wild type strain, was dissociated in strain K1 [20]. Furthermore, *msp* expression is affected by the inactivation of dentilisin [21]; however, the mechanism underlying this phenomenon is yet to be clarified. These reports were indicative of interactions between the pathways regulating the expression of dentilisin and Msp, although the mechanisms regulating the expression of each individual gene remain unknown. In this study, we investigated the gene expression profile of Msp-deficient and dentilisin-deficient *T. denticola* mutants to identify the regulation system involved with the inactivation of these virulence genes, focusing on the regulator proteins.

## Materials and methods

### Strains and culture conditions

*T. denticola* ATCC 35405 [22], *msp*-deficient mutant, *T. denticola* DMSP3 [18], and a *prtP*-deficient mutant, *T. denticola* K1 [20], were maintained in TYGVS medium [23] at 37°C under anaerobic conditions (N_2_: 80%, H_2_: 10%, CO_2_: 10%).

### DNA microarray analysis

RNA was extracted from *T. denticola* ATCC 35405, K1, and DMSP3, as described previously [24]. Briefly, mid-log phase-grown *T. denticola* cells were harvested via centrifugation, and total RNA was extracted using the Trizol reagent (Invitrogen, Carlsbad, CA) according to the manufacturer’s instructions. The resultant samples were treated with Turbo DNase I (Life Technologies Japan Ltd, Tokyo, Japan) twice at 37°C for 30 min to remove genomic DNA contamination. The quality of the RNA was evaluated by TapeStation (Agilent Technologies, Santa Clara, CA).

Microarray analysis was performed using the *T. denticola* 8 × 15 K 1 color system (Hokkaido System Science, Sapporo, Japan). Briefly, the labeled cRNA was synthesized using cDNA as the template with cyanine 3′ CTP using the Low Input Quick-Amp WT labeling kit (Agilent Technologies). The labeled cRNA was hybridized to microarray slides. Microarray image acquisition was performed using the Agilent Microarray Scanner and Agilent Feature Extraction 10. 7. 3. 1. (Agilent Technologies). Differentially expressed genes were determined using a fold-change of at least 2.0, with a significance level of p < 0.05. The microarray data have been deposited in NCBI’s Gene Expression Omnibus [25].

### Real-time reverse transcription-PCR (qRT-PCR) analysis

Total RNA was isolated as described above. cDNA was synthesized using ReverTra Ace (Toyobo, Osaka, Japan). To remove RNA contamination, the cDNA samples were treated with RNaseH (New England Biolabs, Tokyo, Japan) at 37°C for 20 min and then at 65 °C for 20 min. The quantity of the DNA was measured using a Quantus fluorometer (Promega, Tokyo, Japan). For qRT-PCR analysis, the cDNA samples were diluted in RNase-free water. For each well, 1 µl of the cDNA samples was added to a solution containing 10 µl TaqMan Fast Universal PCR master mix (Life Technologies Japan Ltd., Tokyo, Japan), 1 µl gene-specific primers mix (Table 1), and 8 µl RNase-free water. The qRT-PCR analysis was performed in triplicates with the StepOnePlus real-time PCR system (Life Technologies Japan Ltd.). The expression of each gene was normalized to that of the 16S rRNA, an internal control.

**Table 1.** Primers for PCR and qRT-PCR.

Primer	Sequence (5′–3′)
prtPF	AGCTTGGGCGGCTCTT
prtPR	ACTGCATTGGTTAAAACCGCAAAT
prtPP^a^	ACGGCTCCGAATTTG
mspF	GGTAAAGACAGCGTCTACTGCAA
mspR	CCTAAGCTTAAATCAAGTCCGAAAGC
mspP^a^	CCCGCAGCAAACAA
AbrBF	GGATGACCTCCTCATACTTGGAGAT
AbrBR	TCATCTATCAATTCTCCTTGGATACTCATCA
AbrBP^a^	TTGCCGCATCACCCTT
TDE_0127 F	GCAATCGAAAACAAACAAAAATGGGT
TDE_0127 R	GGAGTGGTATTGAGTGCATCTGTTA
TDE_0127 P^a^	CCGCCGCAACCTTA
TDE_0814 F	ATGAAAACGGTAGAAGCTATAAAAAATTGTG
TDE_0814 R	TCTACCCGAGGTGGAATCTCTTTT
TDE_0814 P^a^	CCGGTTTCCGCAATTC
16SF	GCCGATGATTGACGCTGATATAC
16SR	CGGACTACCAGGGTATCTAATCCT
16SP^a^	CTCCCCGCACCTTC

a: Taqman probe

### Measurement of dentilisin activity

*T. denticola* ATCC 35405 and DMSP3 grown in TYGVS medium were collected at mid-log (OD_660_ of 0.5) and stationary phase (OD_660_ of 1.0). The aliquots (0.5 ml) were separated into the bacterial pellet and supernatant via centrifugation at 3,000 × *g* for 10 min. The cells were then washed with phosphate-buffered saline (PBS, pH 7.2) and were resuspended in double the volume (1 ml) of 50 mM Tris-HCl buffer (pH 8.0). The supernatant was filtered to remove the remaining cells and was mixed with the same volume of 100 mM Tris-HCl buffer (pH 8.0). The samples (120 µl) were mixed with 30 µl 5 mM synthetic chromogenic substrate for chymotrypsin [N-Succinyl-Ala-Ala-Pro-Phe *p-*nitroanilide (SAAPFNA)] (Sigma, St. Louis, MO) and incubated for 30 min at 37°C. The reaction was then stopped by the addition of 50 µl 20% acetic acid. The release of *p*-nitroanilide was determined by measuring optical density (OD) at 405 nm using a microplate reader (SpecroraMax M5e, Molecular device, Sunnyvale, CA). The OD of the blank sample was subtracted from the OD of each sample.

### Protein profile of the mutant

Protein profiles of the *T. denticola* wild type strain, K1, and DMSP3 were compared using sodium dodecyl sulfate-polyacrylamide gel electrophoresis (SDS-PAGE) and immunoblotting. *T. denticola* strains were washed with PBS twice and disrupted with a sonicator (Branson, Danbury, CT) at 100 W for 5 min on ice. Insoluble material was removed by centrifugation at 6,000 g for 30 min. Ten µg of sonicates were loaded on 10% SDS-polyacrylamide gel and electrophoresed at 30 mA for 60 min. After electrophoresis, the gel was stained with 2D-silver stain reagent II (Cosmobio, Tokyo, Japan). For immunoblotting, 10 µg of sonicates were electrophorased on the gel, as described above. The electrophoresed protein was transferred to apolyvinylidene fluoride (PVDF) membrane (Immobiline, Merck Millipore, Darmstadt, Germany). The membrane was blocked with PBS containing 3% bovine serum albumin at room temperature for 60 min. After washing with PBS containing Tween 20 (PBS-T), the membrane was incubated with 1/20,000 diluted rabbit anti-*T.denticola* Msp serum [20] at room temperature for 60 min and 1/100,000 diluted horseradish peroxidase-conjugated goat anti-rabbit IgG thereafter. The membrane was washed with PBS-T, developed with enhanced chemiluminescence (ECL) western blotting detection reagents (GE Healthcare UK Ltd., Little Chalfont, England), and photographed using LAS-4000 (GE Healthcare UK Ltd.).

### Statistical analyses

All experiments were repeated at least twice. The results were expressed as mean values with standard deviations. Student’s *t*-test and one-way analysis of variance (ANOVA) with Dunnett’s multiple comparison test were used to determine the differences between groups using GraphPad Prism 7.0d (GraphPad Software, La Jolla, CA). The level of significance for all statistical tests was set at p < 0.05.

## Results

### DNA microarray analysis

To investigate the effect of inactivation of *prtP* and *msp* on the gene expression profile of *T. denticola*, DNA microarray analysis of wild type, K1, and DMSP3 strains were performed. In K1, the expression levels of ParB-like nuclease, DNA-binding protein (TDE_0127), several ABC transporters, peptidase, silent mating-type information regulation (Sir) 2 family of transcriptional regulators, and membrane proteins such as OmpA and lipoprotein were increased (Table 2). On the other hand, the expression of several ABC transporters, MATE family transporters, acetyltransferases, lipoproteins, and Msp was reduced in K1 (Table 3). In DMSP3, the number of genes with increased expression was relatively lower than that in K1. The expression levels of alpha/beta fold family hydrolase, AbrB family transcriptional regulator (TDE_0344), purine nucleoside phosphorylase, and lipoprotein were increased in DMSP3 (Table 4). The expression of the surface antigen BspA was significantly reduced in DMSP3. In addition, genes encoding methyltransferase, methylthioribose kinase, several transporters, tetratricopeptide repeats (TPR), and methylthioribose kinase were downregulated (Table 5). The expression of *msp* in strain K1 was significantly lower than that in the wild type strain (Table 3). In addition, the expression of *prtP* in strain DMSP3 was 0.68-fold lower than that in the wild type strain, although it was not statistically significant (Table 5).

**Table 2.** Upregulated genes in *prtP-*deficient mutant.

Gene		Gene expression fold-change (K1 versus wild type)
TDE1162	ParB-like nuclease	85.78
TDE0471	Hypothetical protein	7.06
TDE0127	DNA-binding protein	5.07
TDE2336	Sodium/dicarboxylatesymporte	4.72
TDE1947	ABC transporter permease	4.40
TDE1584	Lipoprotein	4.16
TDE1946	Hypothetical protein	4.07
TDE0573	Hypothetical protein	3.97
TDE0816	M20/M25/M40 family peptidase	3.90
TDE2495	Hypothetical protein	3.89
TDE1669	Hemolysin	3.84
TDE0815	Hypothetical protein	3.78
TDE0814	Transcriptional regulator	3.58
TDE1948	ABC transporter permease	3.54
TDE1860	Hypothetical protein	3.42
TDE2125	*aat* leucyl/phenylalanyl-tRNA–protein transferase	3.38
TDE2493	Hypothetical protein	3.34
TDE2277	Sir2 family transcriptional regulator	3.22
TDE0122	Dihydrouridine synthase	3.18
TDE2259	Hypothetical protein	3.06
TDE0668	*sufD* FeS assembly protein SufD	3.06
TDE0395	Oligopeptide/dipeptide ABC transporter permease	2.99
TDE0396	Oligopeptide/dipeptide ABC transporter ATP-binding protein	2.97
TDE2494	Hypothetical protein	2.83
TDE2260	Hypothetical protein	2.80
TDE0397	Oligopeptide/dipeptide ABC transporter ATP-binding protein	2.80
TDE0394	Oligopeptide/dipeptide ABC transporter permease	2.71
TDE1723	Hypothetical protein	2.65
TDE1232	Hypothetical protein	2.62
TDE0626	Hypothetical protein	2.60
TDE0500	Hypothetical protein	2.58
TDE1663	OmpA protein	2.57
TDE0569	Hypothetical protein	2.55
TDE1582	*glgA* glycogen synthase	2.47
TDE0741	Hypothetical protein	2.46
TDE1594	Glutamate synthase (NADPH) small subunit	2.41
TDE1593	Fe-hydrogenase	2.38
TDE1664	Hypothetical protein	2.31
TDE0709	*msrA* bifunctional methionine sulfoxide reductase A/B	2.30
TDE0490	Hypothetical protein	2.22
TDE2242	Hypothetical protein	2.20
TDE2204	Na+/H+ antiporter	2.16
TDE0350	MATE family transporter	2.16
TDE0178	Methyl-accepting chemotaxis protein	2.15
TDE2103	Glycosyl transferase	2.14
TDE0833	Lipoprotein	2.13
TDE0078	*grdB*-1 glycine reductase complex selenoprotein GrdB1	2.11
TDE2623	Hypothetical protein	2.10
TDE2257	5ʹ-Nucleotidase	2.10
TDE1854	Nucleotidyltransferase	2.10
TDE2781	ABC transporter ATP-binding protein/permease	2.09
TDE2278	Hypothetical protein	2.08
TDE2163	Hypothetical protein	2.08
TDE0870	Phosphatase/nucleotidase	2.07
TDE0281	Hypothetical protein	2.05
TDE0345	*dmcB* methyl-accepting chemotaxis protein DmcB	2.05
TDE2104	Hypothetical protein	2.03
TDE1914	*ruvB* Holliday junction DNA helicase RuvB	2.02

The expression of the genes was 2-fold higher than that in the wild type (p < 0.05).

**Table 3.** Genes downregulated in *prtP-*deficient mutant.

Gene		Gene expression fold-change (K1 versus wild type)
TDE0650	Hypothetical protein	−16.12
TDE0754	Hypothetical protein	−5.23
TDE0753	Hypothetical protein	−4.74
TDE0985	Oligopeptide/dipeptide ABC transporter periplasmic peptide-binding protein	−4.56
TDE0764	Hypothetical protein	−3.86
TDE0360	ABC transporter ATP-binding protein/permease	−3.02
TDE0359	ABC transporter ATP-binding protein/permease	−3.01
TDE1533	Hypothetical protein	−2.85
TDE2475	MATE family transporter	−2.66
TDE2474	Acetyltransferase	−2.63
TDE1185	Lipoprotein	−2.49
TDE1274	Oligopeptide/dipeptide ABC transporter permease	−2.45
TDE0365	Hypothetical protein	−2.41
TDE0752	Hypothetical protein	−2.39
TDE1735	Hypothetical protein	−2.37
TDE0469	Hypothetical protein	−2.35
TDE0405	Major outer sheath protein	−2.35
TDE0748	Iron compound ABC transporter periplasmic iron compound-binding protein	−2.33
TDE1843	Hypothetical protein	−2.28
TDE2476	*arcC* carbamate kinase	−2.22
TDE0130	Sodium/dicarboxylate symporter	−2.19
TDE0367	Hypothetical protein	−2.17
TDE0131	Hypothetical protein	−2.15
TDE0368	Type I restriction-modification system, S subunit	−2.08
TDE1817	Hypothetical protein	−2.07
TDE0439	Hypothetical protein	−2.06
TDE1259	Amino acid carrier	−2.04
TDE0175	Pyrrolidone-carboxylate peptidase	−2.03
TDE0443	Hypothetical protein	−2.03
TDE0132	Hypothetical protein	−2.01
TDE1640	3-Dehydroquinate dehydratase	−2.00

The expression of the genes was 2-fold lower than that in the wild type (p < 0.05).

**Table 4.** Genes upregulated in *msp-*deficient mutant.

Gene		Gene expression fold-change (DMSP3 versus wild type)
TDE2269	Hypothetical protein	34.81
TDE0343	Alpha/beta fold family hydrolase	3.56
TDE1723	Hypothetical protein	2.74
TDE0344	AbrB family transcriptional regulator	2.58
TDE2268	Hypothetical protein	2.47
TDE0945	Hypothetical protein	2.43
TDE1954	Prevent-host-death	2.32
TDE0946	Hypothetical protein	2.25
TDE0463	Purine nucleoside phosphorylase	2.24
TDE0833	Lipoprotein	2.18

The expression of the genes was 2-fold higher than that in the wild type (p < 0.05).

**Table 5.** Genes downregulated in *msp-*deficient mutant.

Gene		Gene expression fold-change (DMSP3 versus wild type)
TDE2258	Surface antigen BspA	−20.49
TDE0690	tRNA (5-methylaminomethyl-2-thiouridylate)-methyltransferase	−12.84
TDE2782	ABC transporter ATP-binding protein/permease	−12.61
TDE0964	Hypothetical protein	−11.78
TDE0688	Hypothetical protein	−11.01
TDE0689	Methylthioribose kinase	−10.96
TDE0043	TPR (tetratricopeptide repeats)	−10.85
TDE0986	Oligopeptide/dipeptide ABC transporter ATP-binding protein	−9.56
TDE0985	Oligopeptide/dipeptide ABC transporter periplasmic peptide-binding protein	−8.83
TDE1020	Dicarboxylate transporter periplasmic dicarboxylate-binding protein	−6.58
TDE0687	Phosphoribosylaminoimidazole carboxylase catalytic subunit PurE	−5.80
TDE0250	Sodium-dependent transporter	−5.76
TDE0150	Ribonuclease BN-like	−5.54
TDE0251	Tryptophanase	−5.43
TDE0365	Hypothetical protein	−5.21
TDE0988	Oligopeptide/dipeptide ABC transporter peptide-binding protein	−5.02
TDE0982	Dihydroorotate dehydrogenase/oxidoreductase, FAD-binding	−4.69
TDE0366	Type I restriction-modification system, R subunit	−4.66
TDE1559	Hypothetical protein	−4.60
TDE1080	Dimethyladenosine transferase	−3.93
TDE0981	Hypothetical protein	−3.91
TDE1563	Hypothetical protein	−3.91
TDE1209	Phage integrase family site-specific recombinase	−3.81
TDE1322	Hypothetical protein	−3.71
TDE2781	ABC transporter ATP-binding protein/permease	−3.70
TDE0182	ABC transporter ATP-binding protein	−3.47
TDE1234	Hypothetical protein	−3.31
TDE0581	Transglutaminase	−3.30
TDE1562	Hypothetical protein	−3.14
TDE2331	Hypothetical protein	−3.09
TDE1560	YD repeat-containing protein	−3.07
TDE0094	Hypothetical protein	−3.03
TDE1457	Hypothetical protein	−2.98
TDE1458	Hypothetical protein	−2.92
TDE1558	YD repeat-containing protein	−2.78
TDE2125	Leucyl/phenylalanyl-tRNA–protein transferase	−2.51
TDE1561	Hypothetical protein	−2.50
TDE0179	Hypothetical protein	−2.36
TDE0860	16S rRNA pseudouridine(516) synthase	−2.29
TDE0180	Hypothetical protein	−2.27
TDE0367	Hypothetical protein	−2.26
TDE0181	Methyl-accepting chemotaxis protein	−2.25
TDE0733	Hypothetical protein	−2.23
TDE1640	3-Dehydroquinate dehydratase	−2.16
TDE0584	Permease	−2.15
TDE2074	23S rRNA (adenine(2503)-C(2))-methyltransferase RlmN	−2.07
TDE0112	Hypothetical protein	−2.06
TDE1211	ATP-dependent protease ATP-binding subunit HslU	−2.04
TDE0762	*prtP*	−0.68

The expression of the genes was 2-fold lower than that in the wild type (p < 0.05) except that of TDE0762.

### Expression analysis of msp and prtP in the mutants

**Figure 1.**
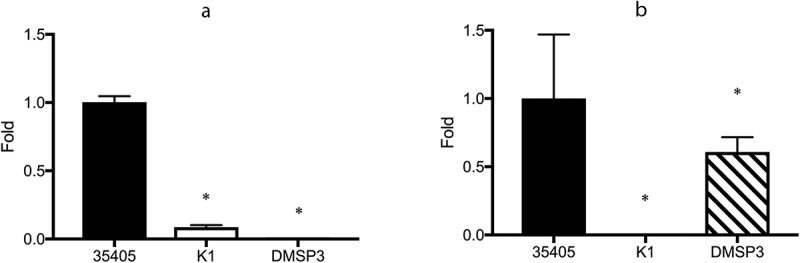
Expression of *msp* and *prtP* determined using qRT-PCR. (**a**) Expression of *msp*. (**b**) Expression of *prtP* (dentilisin); 35405: *T. denticola* ATCC 35405 (wild type), K1: dentilisin mutant, DMSP3: *msp* mutant. The expression of each gene is presented as fold-change over that in the wild type strain. Data are presented as means ± SD (n = 6), and statistically significant differences are indicated using asterisks (one-way ANOVA with Dunnett’s multiple comparison test; *p < 0.05 compared to the wild type).

qRT-PCR was performed to confirm the above observations. Figure 1(a) shows the expression of *msp* in the wild type and mutant strains. In agreement with the −2.35-fold reduction in *msp* expression observed in the microarray assay, *msp* expression was significantly reduced to 8.7% of that in the wild type strain in the dentilisin mutant. Although the expression of *prtP* was somewhat lower in the microarray assay, it was not statistically significant. However, the qRT-PCR assay showed a significant, albeit slight reduction in *prtP* expression in the *msp* mutant (39.3%, Figure 1(b)).

**Figure 2.**
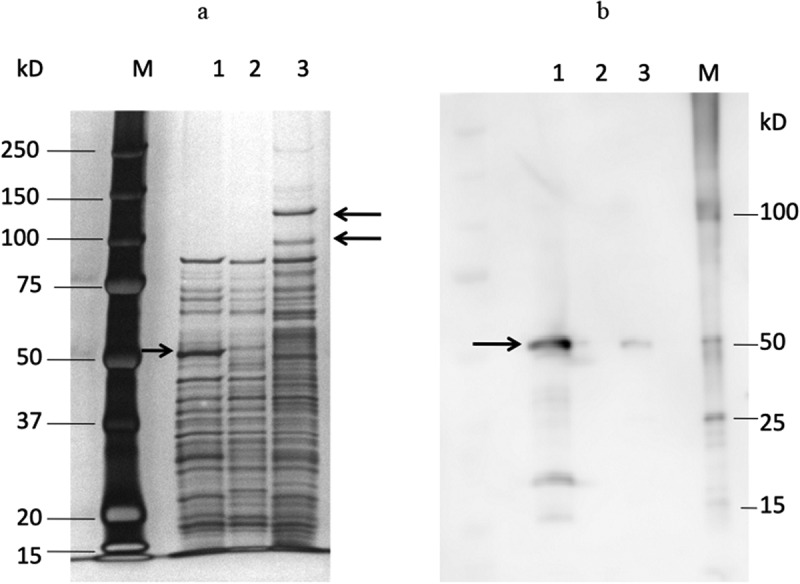
SDS-PAGE and immunoblot analysis of sonicates of *T. denticola* ATCC 35405, DMSP3, and K1. SDS-PAGE analysis (**a**) and immunoblot analysis (**b**).

The reduction in expression was also confirmed at the protein level. The results of the SDS-PAGE analysis of DMSP3 and K1 are shown in Figure 2(a). In strain DMSP3, a 53-kDa band disappeared, while in K1, 100 kDa and 130 kDa bands, which were not detected in the wild type, were observed, along with a smeared band at around 60 kDa. These bands in K1 were detected in the PMSF-treated wild type strain (data not shown), suggesting that these bands were degraded by dentilisin in the process of sample preparation for SDS-PAGE. The results of immunoblotting using an anti-Msp antibody revealed that the density of the Msp band was significantly lower in K1 compared to that in the wild type (Figure 2(b)). These results indicated that the inactivation of *prtP* affects the expression of *msp*.

(**a**) Lane M: Molecular size marker; lane 1: *T. denticola* ATCC 35405 (wild type, 10 µg protein); lane 2: *T. denticola* DMSP3 (*msp* mutant, 10 µg protein); lane 3: *T. denticola* K1 (*prtP* mutant, 10 µg protein). After electrophoresis, the gel was stained with silver stain. Arrow in lane 1 indicates the Msp band and arrows in lane 3 indicate the 100 kDa and 130 kDa bands. (**b**) Lane 1: *T. denticola* ATCC 35405 (wild type, 10 µg protein);lane 2: *T. denticola* DMSP3 (*msp* mutant, 10 µg protein); lane 3: *T. denticola* K1 (*prtP* mutant, 10 µg protein); M: molecular size marker. Msp was detected by 1/20,000 diluted anti-Msp antibody. Arrow in lane 1 indicates the band of Msp.

**Figure 3.**
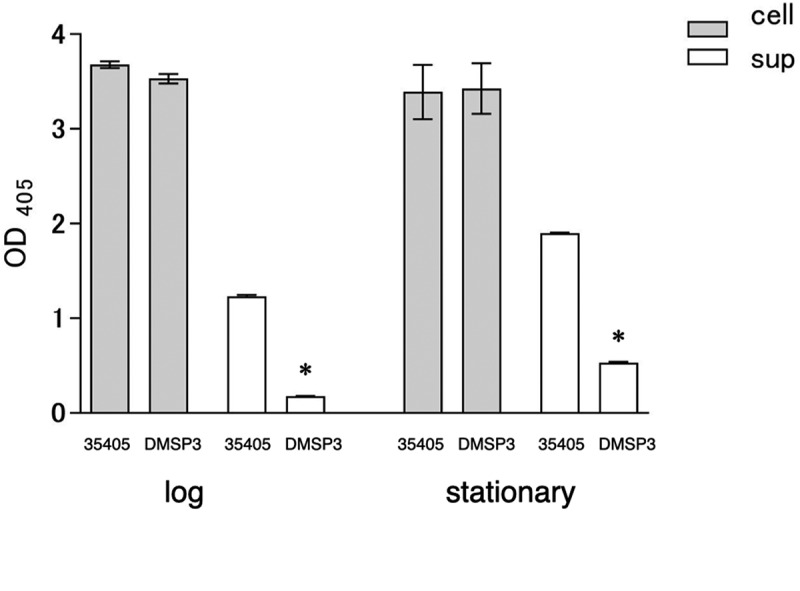
Dentilisin activity of wild type (35405) and *msp-*deficient mutant (DMSP3). Data are presented as means ± SD, and statistically significant differences are indicated by asterisks (student t-test; *p < 0.05 compared to the wild type). 35405: *T. denticola* ATCC 35405, DMSP3: *T. denticola* DMSP3, cell: cells of *T. denticola*, sup: culture supernatant of *T. denticola*, log: log phase, stationary: stationary phase.

Although the expression of dentilisin in the *msp* mutant showed a statistically significant reduction, it was not biologically significant. The dentilisin activity in the *msp* mutant was assayed to clarify its expression. The dentilisin activity assay indicated that the cell-associated dentilisin activity of the *msp* mutant was almost similar in the wild type and mutant strains; however, the activity in the culture supernatant of the mutant was significantly lower than that of the wild type (Figure 3). These results indicated that the expression of the virulence factors was interrelated, as the expression of one was affected by the inactivation of the other.

### Expression of potential transcriptional regulators in the mutants

**Figure 4.**
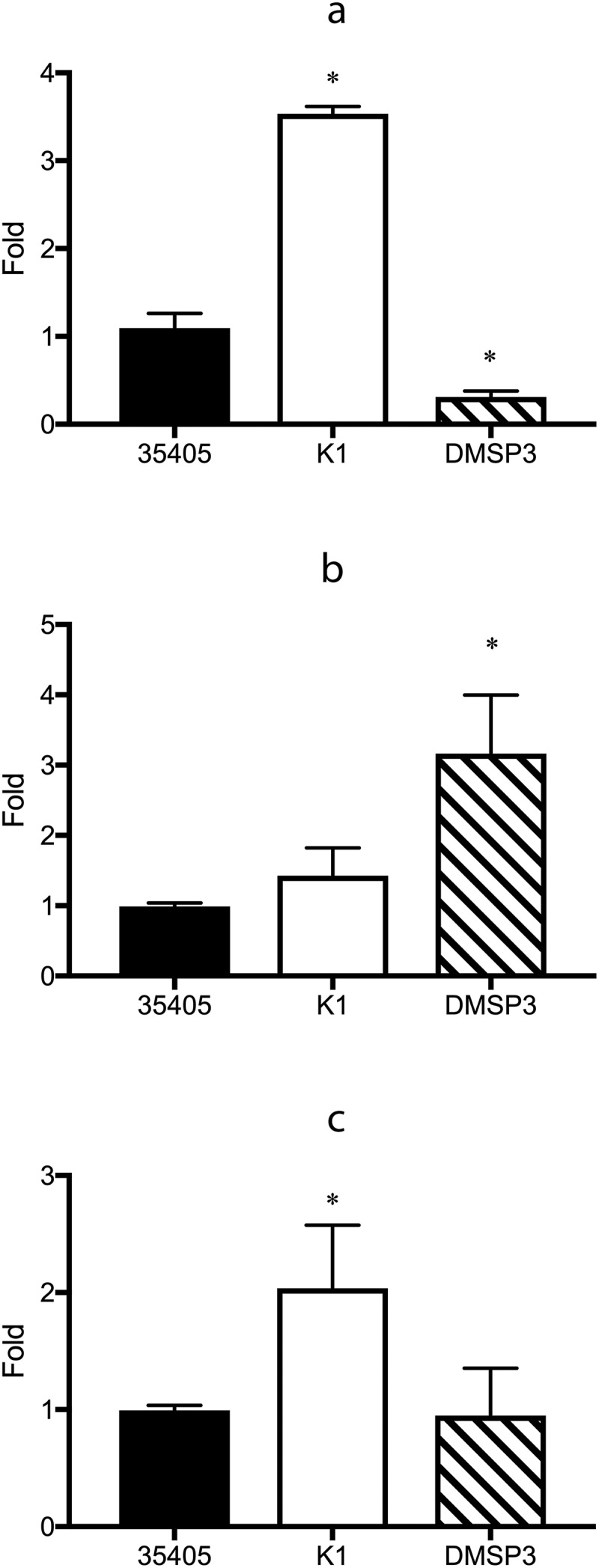
Gene expression of the potential regulator proteins in wild type (35405), *msp-*deficient mutant (DMSP3), and *prtP-*deficient mutant (K1). Gene expression levels were evaluated using qRT-PCR with a Taqman probe. The expression of TDE_0127 (**a**), TDE_0344 (**b**), and TDE_0814 (**c**) are presented as fold-change with respect to that of the wild type strain. Data are presented as means ± SD (n = 6), and statistically significant differences are indicated by asterisks (one-way ANOVA with Dunnett’s multiple comparison test; *p < 0.05 compared to wild type).

The results of microarray and qRT-PCR analyses showed that the inactivation of *msp* affected the expression of *prtP* and vice versa. To clarify this further, the expression of several potential transcriptional regulators, whose expression levels were high in the microarray analysis, were investigated using qRT-PCR. In K1, the expression levels of DNA-binding protein (TDE_0127) and transcriptional regulator (TDE_0814) were significantly higher than those in the wild type strain (Figure 4(a and c)). In the genome sequence of *T. denticola* ATCC 35405, two open reading frames (TDE_0037 and TDE_0344) are annotated as the AbrB family of transcriptional regulators. In DMSP3, the expression of the AbrB family of transcriptional regulators (TDE_0344) was 3.2 times higher than that in the wild type strain, while that of the DNA-binding protein (TDE_0127) was reduced to 28.4% of that in the wild type strain (Figure 4(a and b)).

## Discussion

In the present study, the inactivation of *prtP* affected the expression of *msp* and vice versa. The reduction of *msp* expression in the dentilisin mutant was in agreement with the results of Bian et al. [21], who reported that compared to that in the wild type, the level of total Msp was considerably reduced and that the Msp oligomers were barely detected in the *prtP-*deficient mutant. In the present study, the results of qRT-PCR and immunoblotting indicated that the *msp* expression at both mRNA and protein levels, respectively, was reduced in the *prtP*-deficient mutant. Interestingly, mRNA expression of *prtP* was also reduced in the *msp* mutant. Cell-associated dentilisin activity in *T. denticola* wild type and the *msp* mutant was almost similar, as determined by the activity assay; however, the activity of the *msp* mutant in the culture supernatant was significantly lower than that of the wild type supernatant. Vesicle release has been reported in *T. denticola*, and Msp and dentilisin are located in the vesicle [26]. A recent report indicated that PrtP, PrcA1, and PrcA2 from PacA, and PrcB were enriched in the outer membrane vesicle [27]. The inactivation of Msp or the reduction of *prtP* expression may affect the formation of vesicles containing dentilisin. These results suggested the existence of a regulatory system, which acts in response to the inactivation of *prtP* or *msp* to regulate their expression.

Dentilisin and *msp* mutations altered the mRNA expression profiles of a number of genes with various functions, suggesting the involvement of a gene repressor or activator in regulating gene expression in the mutants. Sigma factors, the two-component system, and the one-component system control bacterial gene expression [28–30]. The two-component system has been studied in the adaptation of *T. denticola* [31]. Regulation by the AtcR LytTR domain-containing response regulator has been reported, and these proteins regulated gene expression in response to environmental conditions such as oxygen levels [32,33]. The regulation by the sigma factors has not been clarified yet. In the one-component system, TroR, which regulates the *troABCDR* operon as a repressor under iron- and manganese-limited conditions, is the only regulatory protein [34,35]. The regulator proteins identified in the previous studies are not reported to regulate the expression of Msp or dentilisin. In the present study, changes in the expression of potential sigma factors and response regulators were not observed. In contrast, the expression level of the DNA-binding protein (TDE_0127) in K1 was 3.54 times higher than that in the wild type. The predicted structure of this protein is similar to that of helix-turn-helix DNA-binding proteins (cd00093, NCBI), which include the HipB protein of *Escherichia coli*, a protein belonging to the xenobiotic response element family of transcriptional regulators. Interestingly, the TDE_0127 mRNA level was reduced in DMSP3. It indicated that TDE_0127 regulates gene expression in response to both *msp* and *prtP* inactivation. TDE_0127 level has been reported to increase under oxidative stress [36]. TDE_0127 may be involved in gene regulation under multiple stress conditions.

In strain DMSP3, a large number of genes were downregulated compared to that in the wild type, while a few genes were upregulated in the strain. The expression of the surface antigen BspA showed a − 20.49-fold reduction. This protein is reported to be involved in coaggregation with *T. forsythia* and adhesion to HEp-2 cells [37]. As Msp is the major surface protein, a regulation system concerning the surface molecule may be involved in this change. Among the genes with increased expression in the *msp* mutant, the AbrB family transcriptional regulator (TDE_0344) showed a 3.16-fold higher expression than that in the wild type strain. On the other hand, the expression of another AbrB family transcriptional regulator, TDE_0037, was not changed in the *msp* mutant. This gene was co-transcribed with ActR-ActS, a member of the two-component system, and the expression of the operon increased during late-stage growth [38]; however, the function was not clear. The function of the proteins coded by TDE_0037 and TDE_0344 might be different, although both have similarities with the AbrB family transcriptional regulator. In *Bacillus subtilis*, AbrB is involved in the control of over 40 different genes nominally expressed or repressed in suboptimal environmental conditions characterized by limited nutrient resources [39,40]. AbrB acts as a repressor and activator in the regulation of post-exponential gene expression in *B. subtilis* and regulates subtilisin expression in *B. subtilis* [40]. In the present study, TDE_0344 was increased in the *msp*-deficient mutant. Dentilisin and Msp organize a complex in *T. denticola* [11], and Msp and PrcA2 interact with the outer sheath [41]. TDE_0344 might be activated by a stimulus sensed by the outer membrane proteins. The amino acid sequence of dentilisin is similar to that of subtilisin [14]. It is possible that the AbrB family transcriptional regulator (TDE_0344) controls the recovery of the inactivated or downregulated genes, including *prtP*, although further analysis is required to verify this hypothesis.

The transcriptional regulator, TDE_0814, showed similarity to HxlR-like helix-turn-helix (pfam01638, DDBJ), which is a DNA-binding protein that acts as a positive regulator of the formaldehyde-inducible hxlAB operon in *B. subtilis* [42]. In the present study, compared to that in the wild type, the expression of several genes increased in strain K1. Possibly, this increased expression reflected the increase in the expression of TDE_0814. The expression of the Sir2 family of transcriptional regulators was increased in K1. Sir proteins are conserved across eukaryotes and are involved in deacetylating several substrates. Sirtuins are an evolutionarily conserved family of NAD (+)-dependent protein deacetylases that function in the regulation of gene transcription, cellular metabolism, and aging [43]. However, their role in the regulation of prokaryotic gene expression is unclear. The Sir2 transcriptional regulator is unlikely to regulate the expression of *T. denticola* genes, including *msp* although further analysis is required.

Taken together, the inactivation of *prtP* or *msp* induced changes in the expression of several genes, including *prtP* and *msp*, indicating that the expression of these virulence factors was interrelated. Several potential DNA-binding proteins or transcriptional regulators, such as TDE_0127, are suggested to be involved in the regulation system, which acts concomitantly with the inactivation of Msp and dentilisin. However, further analysis is required to clarify the DNA-binding activity and the binding site on the DNA.
